# 5T4-specific chimeric antigen receptor modification promotes the immune efficacy of cytokine-induced killer cells against nasopharyngeal carcinoma stem cell-like cells

**DOI:** 10.1038/s41598-017-04756-9

**Published:** 2017-07-07

**Authors:** Xueyang Guo, Hang Zheng, Weiren Luo, Qianbing Zhang, Jingxian Liu, Kaitai Yao

**Affiliations:** 1 Guangdong Provincial Key Laboratory of Cancer Immunotherapy and Guangzhou Key Laboratory of Tumour Immunology Research, Cancer Research Institute, Southern Medical University, Guangzhou, China; 2 Department of Oncology, Nanfang Hospital, Southern Medical University, Guangzhou, China; 3 Department of Pathology, Shenzhen Third People’s Hospital, Shenzhen University, Shenzhen, China; 4 Shenzhen Hospital, Southern Medical University, Shenzhen, China

## Abstract

Relapse and metastasis of nasopharyngeal carcinoma (NPC) are presumably attributed to cancer stem cells (CSCs). In recent years, chimeric antigen receptor (CAR)-modified immune effector cells have been shown to have impressive antitumour efficacy. In this study, we aimed to identify appropriate tumour-associated antigens predominantly expressed on NPC stem cells (NPCSCs) and determine their suitability for CAR-engineered cytokine-induced killer (CIK) cell therapy against NPC. By investigating the expression patterns of potential target antigens (ROR1, 5T4 and CAIX) in NPC, we found that the oncofetal antigen 5T4 was predominately expressed in NPC cell lines and tissues but absent in non-cancerous nasopharyngeal tissues. Moreover, significantly enhanced expression of 5T4 in NPC spheroids revealed its relationship with putative NPCSCs. Hence, we designed a CAR construct (5T4-28Z) specific for 5T4 and generated CAR-transduced CIK cells. Our results showed that the artificial CAR was efficiently expressed on the surface of CIK cells and that no native phenotypes were altered by the gene transduction. Functional assays revealed that 5T4-28Z-CIK cells possessed both CAR-mediated and CAR-independent anti-NPC activity and were capable of efficiently attacking NPC cells, especially NPCSC-like cells *in vitro*, suggesting that they might serve as an attractive tool for developing efficient therapies against NPC.

## Introduction

Nasopharyngeal carcinoma (NPC), a distinctive head and neck malignancy originating from nasopharyngeal epithelium, is highly prevalent in South China and Southeast Asia, with an annual incidence of more than 25 cases per 100,000 individuals^1^. In recent years, the treatment outcomes for NPC have been greatly improved by radiotherapy and combined chemo-radiotherapy. However, the mortality of NPC patients remains high due to local relapse, distant metastasis and therapeutic resistance^2^. One reasonable explanation for this dilemma is the presence of certain tumour cells called cancer stem cells (CSCs) which harbour characteristics typical of stem cells, such as self-renewal, multi-differentiation ability and unrestricted proliferation. CSCs are believed to be primarily responsible for the initiation, expansion, metastasis and recurrence of neoplasms^3^. Thus far, the presence of CSCs has been verified in multiple malignancies including NPC^4–7^, suggesting that cancer may be radically cured by eradicating CSCs.

In recent years, cell-based immunotherapy using cytokine-induced killer (CIK) cells has achieved promising outcomes in treating various malignancies^8^. CIK cells, which are generated *ex vivo* by inducing peripheral blood mononuclear cells (PBMCs) with IFN-γ, anti-human CD3 antibody (OKT3) and IL-2^9^, represent a heterogeneous immune effector cell population mainly composed of CD3^+^CD56^+^ NKT cells. CIK cells exhibit major histocompatibility complex (MHC) unrestricted, antigen-independent cytolytic activity against various tumour cells, including CSC-like cells. This effect is primarily mediated by an interaction between the activating natural killer cell receptors of CIK cells including natural killer group 2 member D (NKG2D) and the corresponding ligands expressed on the surface of tumour cells^10^. In addition, the robust proliferation ability and negligible toxicity of CIK cells makes them desirable candidates for immunotherapy for treating both haematopoietic and solid tumours^11^. However, the clinical therapeutic efficacy is limited primarily due to the moderate activation of CIK cells, which has restricted their extensive application. This insufficient activation is mainly attributed to the nonspecific recognition pattern of CIK cells and immune tolerance resulting from multiple mechanisms that tumours employ to evade immune surveillance^12^. This result suggests that novel therapeutic strategies with superior specificity and efficacy for treating malignancies are imperative.

More recently, adoptive transfusion of T lymphocytes genetically engineered to express chimeric antigen receptors (CARs) specific for membrane antigens on tumour cells has emerged as an effective approach for treating malignancies. CARs consist of an extracellular antigen-binding region, generally a single chain variable fragment (scFv) derived from a monoclonal antibody (mAb), linked to intracellular signalling components comprising a CD3ζ motif alone or in tandem with one or more co-stimulatory domains to provide survival, activating and propagating signals^13^. The artificial constructs innovatively combine the advantages of cellular and humoral immunity in a single fusion molecule to confer on T cells potent and highly specific anti-neoplasm immune responses. Different from intrinsic T cell receptor (TCR)-mediated tumour recognition, CAR-based antitumour effects circumvent the immune evasion mechanisms of tumour cells by virtue of an MHC unrestricted antigen recognition mode. Moreover, CAR gene modification expands the range of potential targets to include not only protein antigens but also carbohydrate and glycolipid antigens that are not recognized by native TCRs^14^. Based on the above advantages, impressive therapeutic efficacy of CAR-mediated cell therapy has been observed in a series of clinical trials, especially those for chronic lymphocytic leukaemia^15^ and acute lymphoblastic leukaemia^16^. However, severe side effects such as on-target/off-tumour effects have also been reported in some studies^17–19^. These effects are mainly attributed to the recognition of target antigens at low expression levels on normal tissues by CARs, prompting researchers to be more rigorous and prudent in the selection of target antigens for CAR-redirected immunotherapy.

Unique tumour antigens restrictively expressed on the surface of malignant cells are essential and indispensable for CAR gene-transduced immune effector cells to target cancer cells or even CSCs^13^. The CSC theory implies that abnormal expression of stemness-associated genes, some of which play vital roles in embryonic development, particularly epithelial to mesenchymal transition (EMT, a key event that generally occurs during embryogenesis and metastasis of epithelial tumours), is one of the most prominent characteristics of CSCs, distinguishing them from other tumour cells^20^. A portion of these genes are selectively expressed at high levels on the membrane of tumour cells, especially CSCs, but are scarcely found in normal tissues. This observation suggests that these genes may serve as potential targets for CAR-redirected CIK cell-based immunotherapy against malignancies, including NPC.

Currently, there are few therapeutic approaches with high specificity and efficacy against NPC. Therefore, our study primarily focused on three intriguing tumour-associated antigens (TAAs), namely, receptor tyrosine kinase-like orphan receptor 1 (ROR1), trophoblast glycoprotein (TPBG/5T4) and carbonic anhydrase IX (CAIX). These TAAs are expressed in a tumour-restricted mode and have been confirmed to be highly correlated with embryogenesis, EMT or stemness features in many malignancies^21–23^. To ascertain their applicability for CAR-redirected CIK cell therapy against NPC, especially NPC stem cell-like cells, we assessed the expression patterns of these TAAs and their relationship with putative CSCs in NPC. We found that 5T4 might serve as a suitable target antigen. Based on this finding, we engineered CIK cells with an anti-h5T4 CAR construct, identified the phenotypic characteristics of these artificial CAR-CIK cells and evaluated their efficacy against NPC cell lines and putative CSCs *in vitro*.

## Results

### Expression of ROR1, 5T4 and CAIX in NPC cell lines

**Figure 1:**
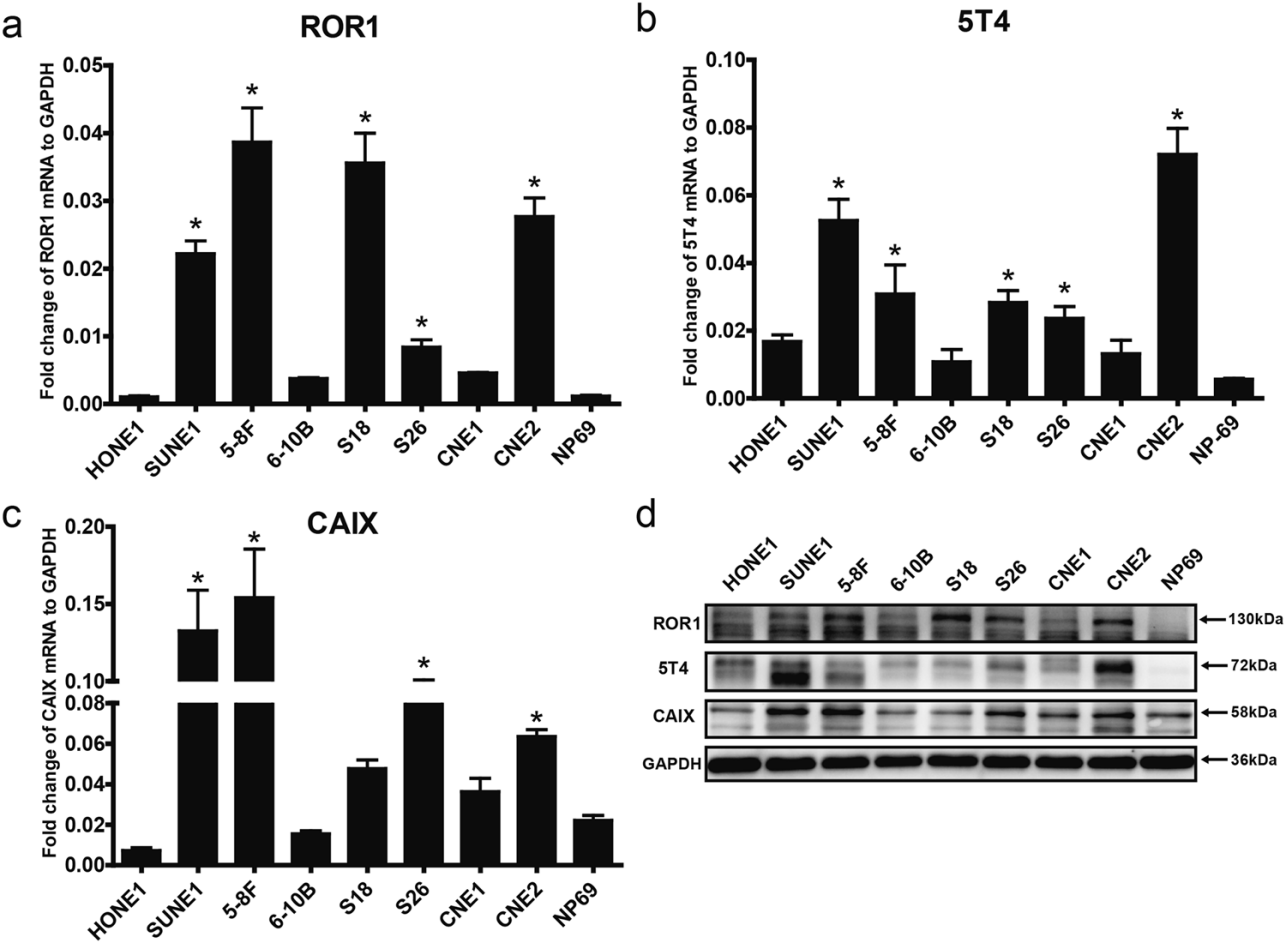
Expression of ROR1, 5T4 and CAIX in NPC cell lines. The mRNA expression levels of ROR1 (**a**) 5T4 (**b**) and CAIX (**c**) in eight NPC cell lines and the normal nasopharyngeal epithelial cell line NP69 were measured using RT-qPCR and normalized to GAPDH. Values are shown as the mean ± SD of triplicate samples. * indicates *p* < 0.05 (one-way ANOVA) in comparison with NP69 cells. (**d**) The protein expression of ROR1, 5T4 and CAIX in the same nine cell lines was probed using WB with specific antibodies. GAPDH was used as a loading control. The molecular weight of each protein is marked on the right. The results shown are representative of three independent experiments.

To determine whether ROR1, 5T4 and CAIX are suitable for CAR-CIK cell-based immunotherapy against NPC, the mRNA expression of these genes in eight NPC cell lines was first evaluated using real-time quantitative PCR (RT-qPCR). There are two variants of the *ROR1* transcript. Splice variant 1 represents the longer transcript and encodes the complete protein, whereas variant 2 comprises a shorter coding region. The resulting isoform 2 lacks the tyrosine kinase domain, compared with isoform 1^24^. We measured the expression of both variants using specific primer pairs. As shown in Fig. 1a, five NPC cell lines (SUNE1, 5–8F, S18, S26 and CNE2) had substantially higher *ROR1* expression levels than the normal nasopharyngeal epithelial cell line (NP69), but all NPC cell lines showed considerably lower *ROR1* transcription levels (<25fold) than those of the housekeeping gene, *GPADH*. Likewise, the *5T4* gene also has two transcript variants, but both of them encode the same protein. RT-qPCR detection revealed that the *5T4* transcription levels in the same five NPC cell lines were over four times higher than that of the NP69 cell line (Fig. 1b). Moreover, four of the five NPC cell lines (except S18) obviously expressed more *CAIX* mRNA than the NP69 cell line (Fig. 1c).

Subsequently, to analyse whether the mRNA transcript levels were in accord with the protein expression, the relative protein expression levels of the target genes in the same eight NPC cell lines were assessed using western blotting (WB) (Fig. 1d). Specific antibody detection of protein lysates from the NPC cell lines showed that ROR1 was an approximately 130 kDa protein expressed at higher levels in four of the NPC cell lines (5–8F, S18, S26 and CNE2) than in the NP69 cell line, in which the ROR1 protein was barely detected. Likewise, in a 5T4 WB analysis, a strong band of 72 kDa was observed in SUNE1 and CNE2 cell lines but undetectable in the NP69 cell line. Furthermore, a 58 kDa CAIX molecule was found to be expressed at higher levels in four of the NPC cell lines (SUNE1, 5–8F, S26 and CNE2) than the others. Notably, NP69 cells also expressed detectible levels of CAIX, although to a lesser extent, suggesting that expression of this gene might not be restricted to NPC cells.

### Expression of ROR1, 5T4 and CAIX in NPC tissues

**Figure 2:**
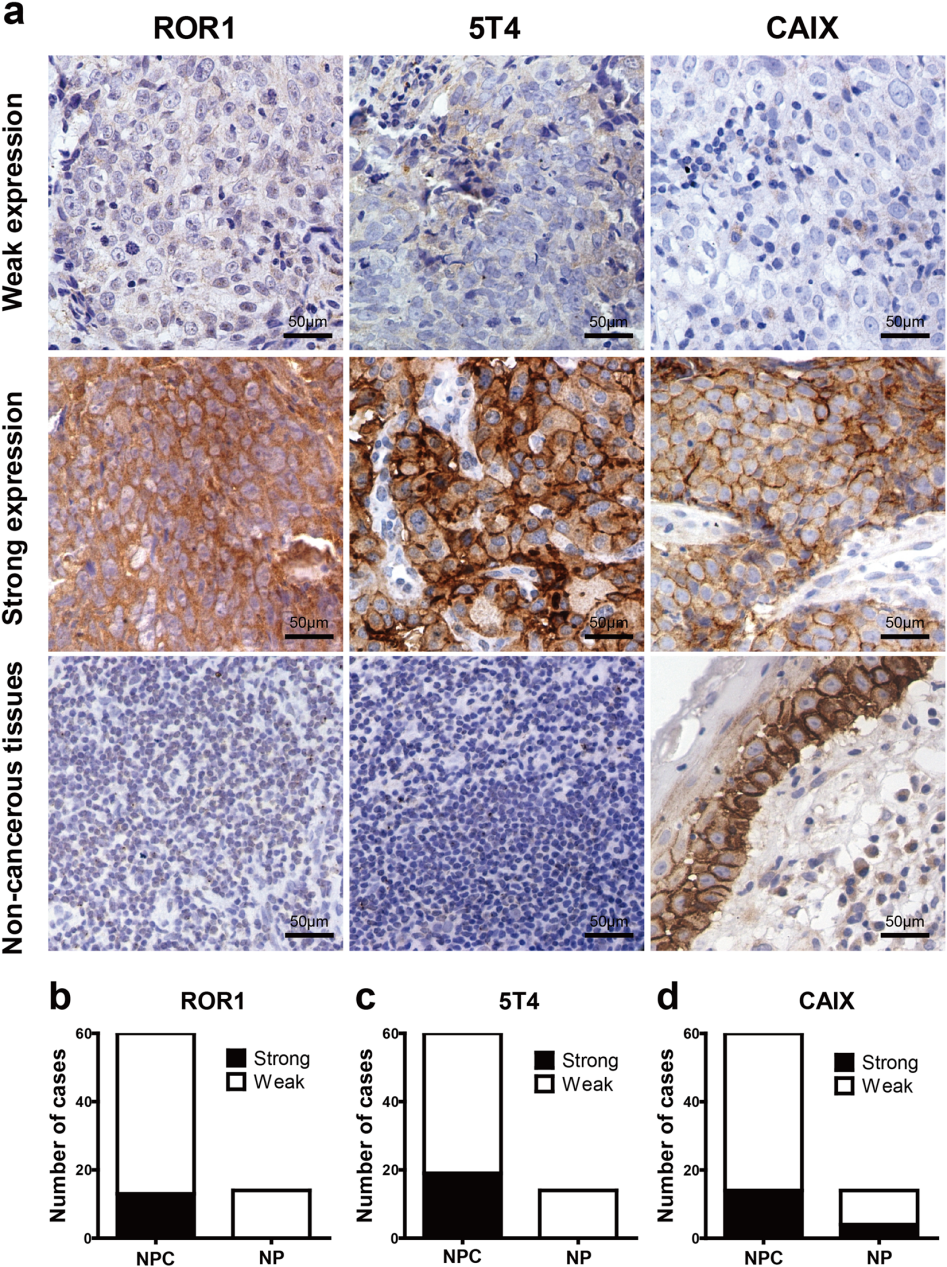
Expression of ROR1, 5T4 and CAIX in NPC tissues. (**a**) The expression patterns of ROR1, 5T4 and CAIX in NPC tissues were determined using IHC with the corresponding antibodies. Representative images of weak expression (score ≤ 4), strong expression (score ≥ 6) and the negative control for each gene are shown. A detailed description is provided in the Methods section. The number of NPC cases (n = 60) and non-cancerous tissues (n = 14) with strong or weak staining of ROR1 (**b**), 5T4 (**c**) and CAIX (**d**) are shown in each histogram.

To further evaluate the feasibility of targeting these antigens in anti-NPC immunotherapy, we studied their expression patterns in NPC and inflammatory nasopharyngeal tissues using immunohistochemistry (IHC) (Fig. 2a). The staining results showed that ROR1, which was predominately located on the membrane of tumour cells, was strongly expressed in 13 of 60 (21.7%) paraffin-embedded NPC specimens. In contrast, none of the total 14 nasopharyngeal epithelial tissues exhibited positive ROR1 staining (Fig. 2b). However, unexpectedly, the following statistical analysis revealed that there was not a significant difference between ROR1 expression in NPC and non-cancerous epithelium (*p* = 0.055). We supposed that this might partly be due to the relatively small sample size. On this account, ROR1 was still involved in the following steps. Similarly, strong membrane staining of 5T4 was observed in 19 of 60 (31.7%) NPC samples, which was significantly more than that in the nasopharyngeal epithelial tissues (*p* < 0.05), where no case of positive 5T4 expression was observed (Fig. 2c). This result was in accord with the 5T4 mRNA and protein expression levels in NPC cell lines, indicating that the 5T4 molecule was selectively expressed on the surface of NPC cells and might serve as a target for immunotherapy against NPC. Likewise, CAIX was found to be highly expressed in 14 of 60 (23.3%) cases and mainly located on the membrane of neoplastic cells. However, positive CAIX staining was also observed in 4 of the 14 (28.6%) non-cancerous epithelial tissues (Fig. 2d), suggesting that this gene was not restrictively expressed in NPC cells and might not be suitable for targeted therapy against NPC. Therefore, CAIX was excluded from further studies.

### 5T4 exhibits enhanced expression in NPC spheroids and is correlated with stemness features of NPC cells

**Figure 3:**
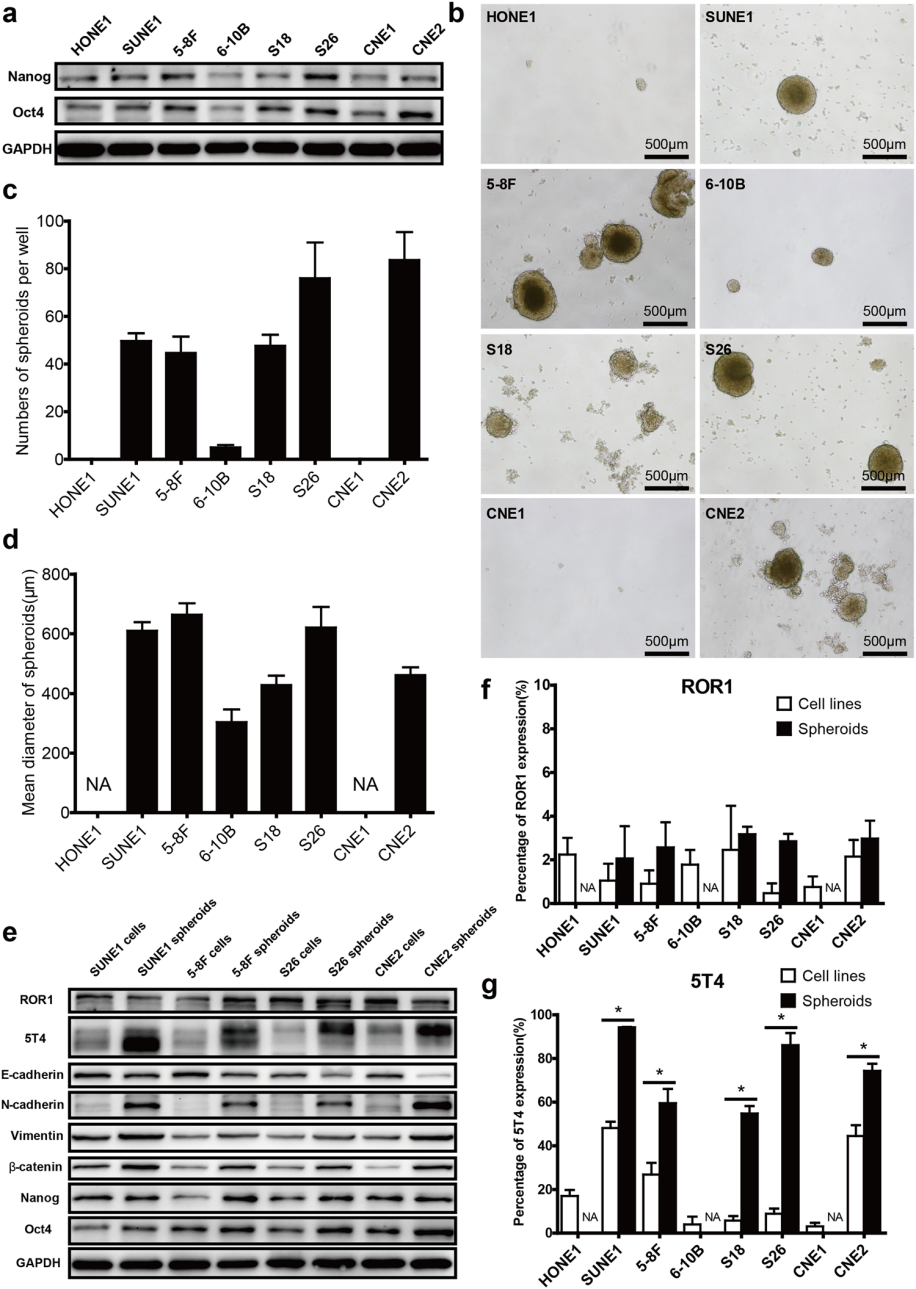
5T4 expression is correlated with stemness features in NPC. (**a**) WB analysis of Nanog and Oct4 expression in eight NPC cell lines was performed through staining with the corresponding antibodies. The results shown are representative of three independent experiments. (**b**) The self-renewal capability of eight NPC cell lines was assessed with sphere formation assays. NPC cells were seeded at a density of 1,000 cells per well in triplicate and induced to spheroids. A typical image of each type of spheroid on day 7 of culture is presented. (**c**) The number of each type of NPC spheroid was calculated and is presented as the mean ± SD of triplicate wells. (**d**) The diameter of each type of NPC spheroid was measured, and the results are displayed as the mean ± SD of five randomly selected spheroids. The experiment was repeated three times. (**e**) The expression levels of ROR1, 5T4 and a series of EMT and stemness-associated markers in NPC cell lines and the resultant spheroids were examined using WB with specific antibodies. The results shown are representative of three independent experiments. Membrane expression of ROR1 (**f**) or 5T4 (**g**) on NPC spheroid cells and their parental cells was analysed using FACS with APC-conjugated specific antibodies or matched isotype control antibodies, respectively. Data are shown as the mean ± SD of triplicate experiments. NA: not available. * indicates *p* < 0.05 (two-tailed Student’s t-test).

To correlate target gene expression and the intrinsic stemness features of NPC cells, the expression levels of stem cell markers (Nanog and Oct4) in eight NPC cell lines were assessed using WB. As shown in Fig. 3a, five NPC cell lines (SUNE1, 5–8F, S18, S26 and CNE2) exhibited higher expression levels of Nanog and Oct4 than the other cell lines, indicating superior stemness. Subsequently, by examining the self-renewal capability of eight NPC cell lines in sphere formation assays, the same five NPC cell lines were found to generate more and larger spheroids than the other cell lines which barely formed spheroids after seven days of cultivation (Fig. 3b-d). Notably, the expression levels of 5T4 and ROR1 proteins were higher in some or all of the five NPC cell lines, suggesting a potential correlation with stemness characteristics in NPC. Furthermore, the target gene expression levels between NPC cell lines and their spheroids were compared using WB and fluorescence-activated cell sorting (FACS). As shown in Fig. 3e, no alteration in ROR1 expression was observed in WB assays after the NPC cells were induced to spheroids, while the FACS assay showed that the same low staining of ROR1 on the surface of spheres and parental cells was maintained (Fig. 3f). These data implied that ROR1 might not be an eligible antigen for targeted immunotherapy against NPC. In contrast, both WB (Fig. 3e) and FACS (Fig. 3g and Supplementary Fig. S1) revealed that the expression of 5T4 protein in all NPC spheroids was significantly increased in comparison with their parental cells (*p* < 0.05). In addition, all spheroids showed down-regulation of the epithelial cell marker E-cadherin and up-regulation of EMT-related molecules (N-cadherin and Vimentin), β-catenin (a key molecule in the Wnt signalling pathway) and stemness indicators (Oct4 and Nanog) compared with the parental cells (Fig. 3e), indicating superior motility, metastatic ability and CSC characteristics. These findings implied that 5T4 expression might be closely associated with putative CSCs in NPC. Therefore, targeting 5T4 might possibly eliminate the NPC cells that highly expressed 5T4 and even CSC-like cells.

### Generation of CAR gene-modified CIK cells specific for human 5T4 oncofetal antigen

**Figure 4:**
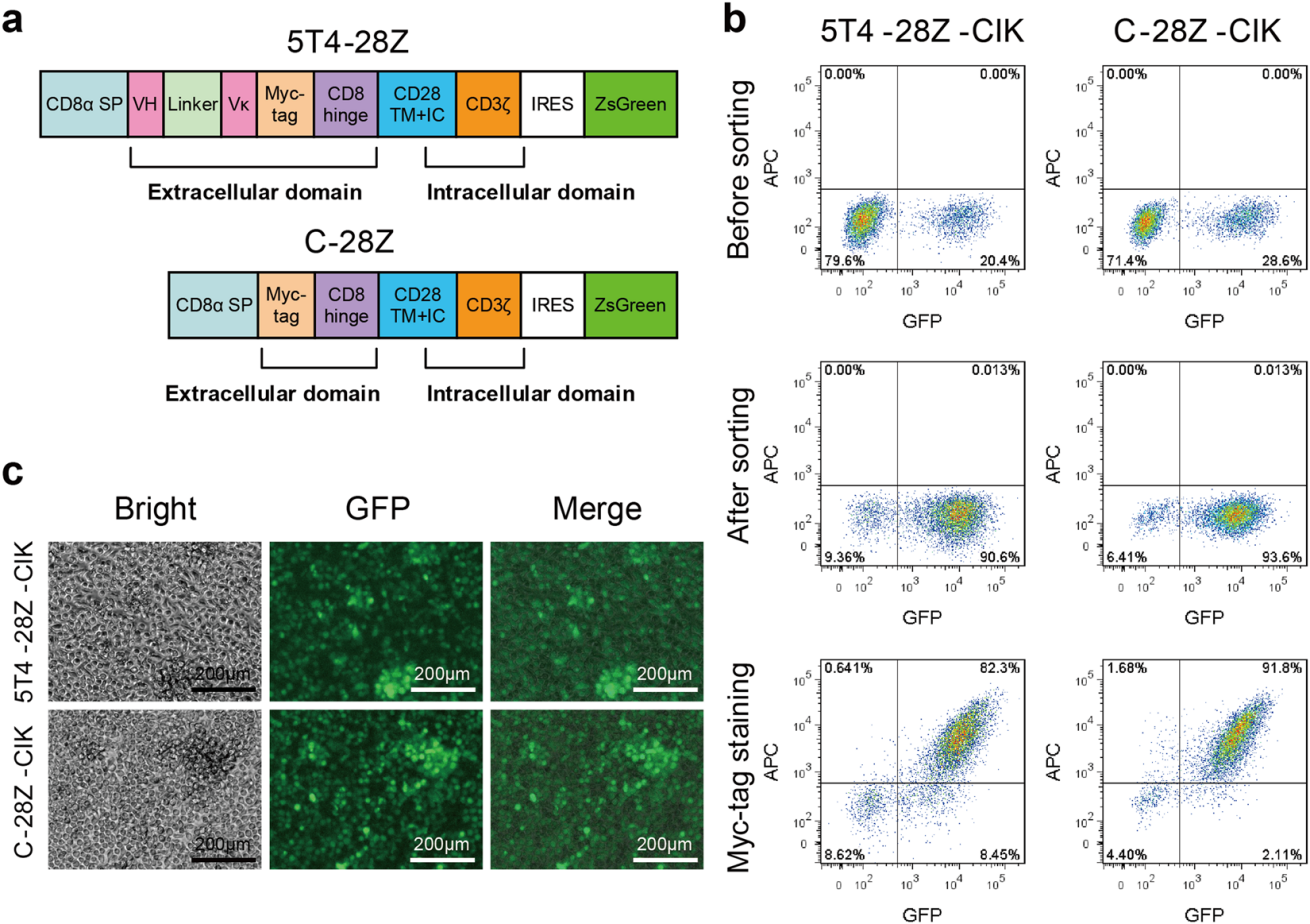
Generation and identification of CAR gene-modified CIK cells. (**a**) Schematic representation of the anti-h5T4 CAR construct and the control CAR construct. The 5T4-28Z CAR contained, in sequence from the N-terminus to the C-terminus, a CD8α signal peptide (SP), an anti-h5T4 scFv comprising the variable regions of the heavy chain (VH) and light chain (Vκ) fused with a linker, a myc-tag sequence, a CD8α hinge, the CD28 transmembrane (TM) and intracellular (IC) regions followed by the CD3ζ signalling domain. The control CAR comprised a sequence identical to 5T4-28Z except without the scFv region. ZsGreen was used as a reporter gene. (**b**) CIK cells were generated as mentioned in the Methods section. They were transduced with CAR-encoded lentivirus and sorted using FACS according to the GFP expression. The surface expression of CARs on CIK cells was verified using FACS with an Alexa Fluor^®^ 647-conjugated myc-tag antibody. As controls, matched isotype antibody staining was incorporated. Flow plots are representative of quintuplicate cultures. (**c**) Brightfield and fluorescence images of sorted 5T4-28Z CAR- or C-28Z CAR-expressing CIK cells were captured. Representative images and merged images are displayed.

A human 5T4-specific CAR construct was generated in a pHAGE-fullEF1α-MCS-IRES- ZsGreen lentiviral vector (Fig. 4a). This construct, designated 5T4-28Z, contained in sequence a CD8α signal peptide (SP), an scFv specifically recognizing 5T4 with high affinity, a myc-tag sequence, a CD8α hinge and the CD28 transmembrane and intracellular region followed by the CD3ζ signalling domain. A control CAR construct consisting of a sequence identical to that of 5T4-28Z but without the scFv region was designated C-28Z. Then, CAR-encoded lentivirus was produced, concentrated and titrated. To obtain CAR-engineered CIK cells, PBMCs from healthy donors were induced to CIK cells in the presence of IFN-γ, CD3mAbs, CD28 mAbs and IL-2 as mentioned above and transduced with CAR-encoded lentivirus three days after stimulation. The mean transduction efficiency was approximately 25%, monitored on day 7 of culture (Fig. 4b). Then, the CAR-expressing CIK cells were enriched to more than 90% purity using FACS according to the expression of green fluorescent protein (GFP) encoded by the vector (Fig. 4c). In addition, surface localization of the CAR on CIK cells was verified by staining with an Alexa Fluor^®^ 647-conjugated myc-tag antibody, because almost all the GFP-positive cells were determined to express the myc-tag.

**Figure 5:**
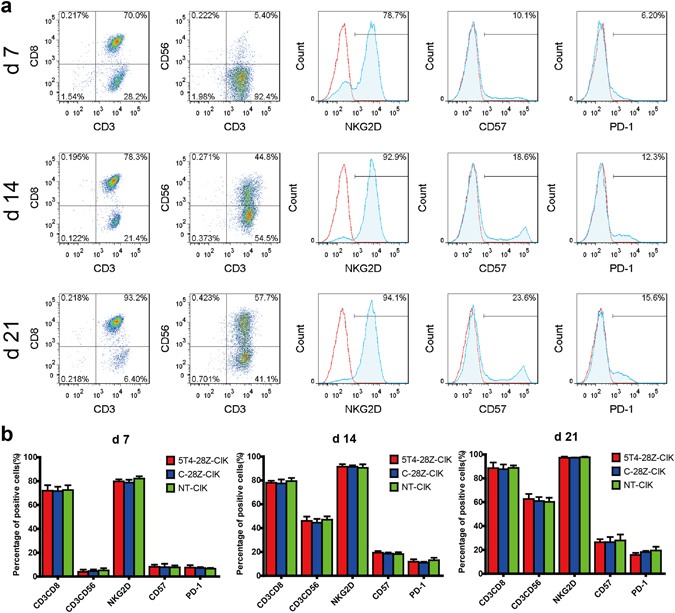
CIK cells maintain their original phenotypes after transduction of CAR genes. The expression of CD3, CD8, CD56, NKG2D, CD57, and PD-1 on CAR-engineered CIK cells and NT-CIK cells was monitored weekly using FACS with diverse fluorochrome-conjugated antibodies or matched isotype control antibodies. (**a**) Characteristic flow plots showing the expression of phenotypic markers in 5T4-28Z-CIK cells on day 7, 14, and 21 of cultivation are presented. (**b**) Histograms show the mean values ± SD of three independent experiments.

To investigate whether the CAR gene modification might affect the phenotypic characteristics of CIK cells, the expression of CD3, CD8, CD56, NKG2D, CD57, PD-1 on CAR-expressing CIK cells and non-transduced CIK (NT-CIK) cells were assessed weekly using FACS (Fig. 5a). The staining results showed that the percentage of CD3^+^CD8^+^ and CD3^+^CD56^+^ populations, which were the critical subsets of CIK cells^25^, kept increasing from the beginning of cultivation and reached 88.7 ± 2.1% and 60.2 ± 3.5% on day 21, respectively. Similarly, NKG2D, one of the most important activating natural killer receptors for CIK cells^9^, was maintained a high expression level (82.1 ± 1.9%~97.6 ± 0.5%) at all time points. In contrast, the replicative senescence marker CD57 and the T cell exhaustion indicator PD-1^26^ remained at a low expression level on CIK cells even after three weeks of culture (27.9 ± 5% and 19.4 ± 3.2%, respectively). These results indicated that the cells had neither proceeded to terminal differentiation nor reached exhaustion throughout the entire culture period. Notably, there was no apparent difference in phenotypes between the CAR-engineered CIK cells and the non-transduced CIK cells (*p* > 0.05), demonstrating that CAR gene modification had not altered the typical phenotypes of the CIK cells (Fig. 5b).

### 5T4-specific CAR gene modification promotes the anti-NPC activity of CIK cells in a 5T4-dependent manner

**Figure 6:**
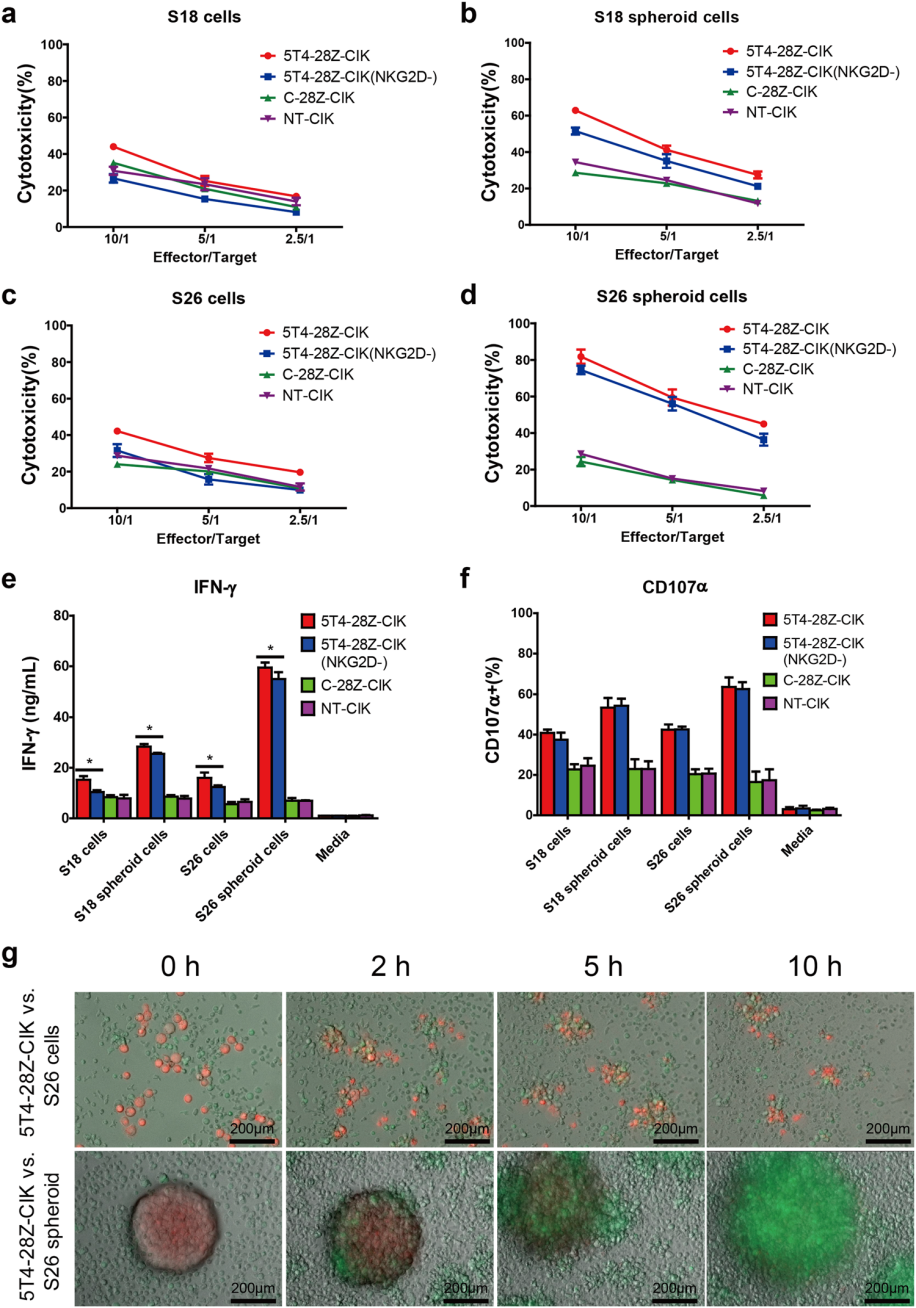
5T4-28Z CAR gene modification promotes the anti-NPC activity of CIK cells in a 5T4-dependent manner. Target cells consisted of 5T4 low-expressing S18 and S26 cells and their spheroid cells with an intermediate or high level of 5T4 expression. Effector cells included 5T4-28Z-CIK, 5T4-28Z-CIK (NKG2D-), C-28Z-CIK and NT-CIK cells. (**a–d**) Briefly, 1 × 10^4^ target cells per well were co-cultured with different effector cells at E/T ratios of 10/1, 5/1 and 2.5/1 for 4 h. The cytotoxicity of effector cells was examined with LDH release assays. Values in the line graphs represent the mean ± SD of three parallel wells. (**e**) Then, 2 × 10^4^ target cells per well were co-incubated with 1 × 10^5^ effector cells per well for 24 h. Effector cells cultured alone in medium served as the negative control. IFN-γ production of the effector cells was assessed using ELISAs. Values in the histogram represent the mean ± SD of three parallel wells. (**f**) In addition, 2 × 10^5^ target cells per well were co-cultured with 1 × 10^6^ effector cells per well for 5 h in the presence of 1 × Protein Transport Inhibitor Cocktail and anti-human CD107α-APC antibody or IgG isotype control. Effector cells cultured alone served as the negative control. Degranulation of effector cells was evaluated using FACS. Values presented in the histogram represent the mean ± SD of triplicate samples. (**g**) S26 cells or S26 spheroids labelled with CM-Dil were co-cultured with 5T4-28Z-CIK cells and monitored using an inverted fluorescence microscope with climate control. Images captured intermittently are displayed. The results are representative of at least three independent experiments. * indicates *p* < 0.05 (one-way ANOVA).

To ascertain whether 5T4-28Z-engineered CIK cells were capable of specifically recognizing and eliminating 5T4-positive NPC cells while maintaining their original antitumour activity, a series of functional assays were performed in which different effector cells were challenged in various ways with 5T4 low-expressing S18 and S26 cells as well as their spheroid cells that exhibited intermediate or high levels of 5T4 expression. First, the cytolytic activity of 5T4-28Z-CIK, C-28Z-CIK and NT-CIK cells at varying effector to target (E/T) ratios was measured with a lactate dehydrogenase (LDH) release assay. As shown in Fig. 6a–d, all effector cells exhibited dose-dependent cytotoxicity. As expected, the NT-CIK cells exerted basal cytotoxic effects on diverse target cells with no apparent difference at all E/T ratios, indicating that their cytolytic activity was unrelated to the 5T4 expression on the surface of NPC cells. However, the 5T4-28Z-redirected CIK cells exhibited varying levels of cytotoxicity enhancement when exposed to different target cells, whereas the C-28Z-CIK cells showed cytotoxicity comparable to NT-CIK cells. These data indicated that CAR gene modification endowed CIK cells with superior and specific anti-neoplasm activity in an antigen-dependent manner. Moreover, blocking NKG2D with a specific neutralizing antibody resulted in a significant inhibition of 5T4-28Z-CIK cell-mediated lysis of all target cells, highlighting that the 5T4-28Z-CIK cells retained their native cytolytic capability partly by relying on NKG2D.

Next, to confirm whether cytotoxicity was accompanied by cytokine release, the IFN-γ production of different effector cells was examined with enzyme-linked immuno-sorbent assays (ELISAs) after 24 h of co-cultivation with target cells. As displayed in Fig. 6e, all effector cells spontaneously secreted a negligible amount of IFN-γ when cultured alone. However, exposure to target cells led to considerably higher levels of IFN-γ secretion by 5T4-28Z-CIK cells than by C-28Z-CIK cells or NT-CIK cells, both of which showed similar IFN-γ production levels. Moreover, surface 5T4 expression on target cells was positively correlated with the IFN-γ production of 5T4-28Z-CIK cells, showing a dose-dependent mode. In particular, masking of NKG2D significantly inhibited the IFN-γ release of 5T4-28Z-CIK cells. This reflected that this receptor plays a role in IFN-γ secretion, which was not interfered with by CAR gene transduction.

In addition, to test the surface expression of lysosomal-associated membrane protein-1 (LAMP1) on effector cells exposed to diverse target cells, a degranulation assay was performed by staining with a CD107α-APC antibody (Fig. 6f). Likewise, similar results were observed, except neutralizing NKG2D failed to alter the proportion of the CD107α^+^ population in 5T4–28Z-CIK cells. This presumably was because cytotoxic granule secretion triggered by CAR redirection was potent enough to cover up the NKG2D-associated degranulation.

Taken together, these findings revealed that the 5T4-28Z CAR-transduced CIK cells possess both CAR-mediated and CAR-independent immune activity against NPC cells.

### Anti-h5T4 CAR-engineered CIK cells efficiently eliminate NPC cells and their spheroids *in vitro*

Furthermore, to visualize the recognition and attack processes of CAR-redirected CIK cells against NPC cells and their spheroids, S26 spheroids or the parental cells were stained with a red fluorescent dye and subsequently co-cultured with 5T4-28Z-CIK cells and monitored using an inverted fluorescence microscope with climate control (Fig. 6g). Time-lapse images revealed that the S26 cells were gradually surrounded by the 5T4-28Z-CIK cells after co-incubation, leading to shrinkage, lysis and release of the cellular content of S26 cells. In another group, the GFP-expressing effector cells increasingly gathered around the CM-Dil-labelled spheroid, encased it and caused it to dissociate, which was accompanied by the dye fading away till it disappeared. Eventually, the spheroid was completely replaced by a cluster of effector cells (Supplementary Videos S1, S2). These results illustrated that the anti-h5T4 CAR-transduced CIK cells were capable of eliminating both 5T4 weakly staining NPC cells and 5T4 highly expressing NPC stem cell-like cells *in vitro*.

## Discussion

In this study, three TAAs (ROR1, 5T4, CAIX) were screened to assess their expression patterns in NPC cells and their potential relationship with NPC stem cell-like cells. However, unlike breast cancer^27^ and ovarian cancer^21^, we found that ROR1 showed limited distribution in NPC, and its expression did not seem to be correlated with putative CSCs. In addition, our research revealed that CAIX was not expressed in an NPC-restricted manner. This result was in agreement with a previous study applying autologous anti-CAIX CAR-T cell therapy to treat metastatic renal cell carcinoma in which severe liver toxicity was observed presumably due to the reactivity of engineered T cells against CAIX expressed on epithelial cells lining the bile ducts^17^. Therefore, we excluded ROR1 and CAIX from the candidates for target antigens.

However, in contrast to the former two antigens, the oncofetal antigen 5T4 displayed varying expression levels in almost all NPC cells, but was absent in NP69 cells. The gene transcription levels were approximately in accord with protein expression levels, although minor variations existed presumably due to post-transcriptional regulation of gene expression. Moreover, the predominant membrane localization of 5T4 on NPC cells (Supplementary Fig. S2) and its selective expression on NPC tissues further indicated that it can be applied for CAR-redirected anti-NPC immunotherapy. This finding prompted us to further ascertain whether 5T4 expression was associated with putative CSCs in NPC.

Oct4, also known as POU5F1, is a member of the POU domain transcription factor family. This molecule, together with Sox2, is crucially involved in self-renewal of undifferentiated embryonic stem cells (ESCs)^28^. In addition, Nanog is a critical molecule for maintaining the pluripotency of ESCs. Our study revealed that five of the eight NPC cell lines with stronger expression of Oct4 and Nanog generated more and larger spheroids in sphere formation assays (a promising approach for enriching CSCs as well as for identifying the self-renewal function of diverse cells^29^). This indicated that these NPC cell lines comprised more putative CSCs. Especially, higher expression levels of 5T4 were observed in SUNE1 and CNE2 cell lines, both of which possessed superior stemness features. Furthermore, compared with the parental cell lines, significantly enhanced surface 5T4 expression was observed in all spheroids. Based on these confirmatory results, we reasoned that 5T4 expression was correlated with the putative CSCs in NPC. In addition, a previous study revealed that CD133, a reliable biomarker for identifying CSCs in NPC^30^, showed comparably low expression levels in both 5T4^hi^ and 5T4^low^ cell populations in non-small cell lung cancer (NSCLC)^22^. Moreover, the study also revealed that 5T4-positive cells were enriched in CD44^+^CD24^−/low^ active/proliferating CSC-like populations and expression of 5T4 represented an undifferentiated state and high tumourigenesis in NSCLC. However, thus far, no data have shown that the CD44^+^CD24^−/low^ populations represented CSCs in NPC. Thus, more evidence is required to further illuminate the functional role of 5T4 and its relationship with CSCs in NPC.

We designed a 5T4-28Z CAR construct to engineer CIK cells for 5T4-targeted therapy against NPC. The variable regions of heavy chain (VH) and light chain (Vκ) derived from a mouse mAb^31^ specific for human 5T4 were fused with a universal linker to fold into their native configuration. The resulting scFv was verified to be capable of specifically binding to the membrane-proximal region of the human 5T4 molecule with high affinity^32–34^. A myc-tag was linked to the C-terminus of scFv for identification of CAR expression on the cell membrane. By referring to the CAR design in the study of Lanitis E *et al*.^35^, we also incorporated the extracellular fragment of CD8α into the CAR structure as a hinge that bridged the scFv and the intracellular signal domain to confer CAR flexibility. This module was believed to contribute to an optimal binding of CAR to target antigen because evidence has shown that the activation level of CAR-T cells depended on both the length of the extracellular non-signalling region and the distance between the epitope and the target cell membrane^36^. For example, CARs binding to epitopes residing close to the N-terminus of antigens seemed to function well in the absence of hinges, whereas CAR-T cells recognizing epitopes located on the membrane-proximal region of target cells tended to exert enhanced activity with the help of longer spacer regions^34^. However, thus far, little knowledge of the function of the hinge region has led to mostly empirical design of CARs and no standardized rules have been developed yet. Additionally, studies involved in the intracellular signalling domain of CARs are in the spotlight and have attracted great interest. The initial “first generation CARs” failed to trigger an effective antitumour immune response *in vivo* and tended to undergo apoptosis or anergy, primarily due to a lack of co-stimulatory signals^37^. On this account, one or more co-stimulatory molecules, such as CD28, CD134 (OX40), CD137 (4-1BB) and ICOS, were integrated into the first generation CARs to endow them with more potent cytolytic activity and enhanced expansion capability as well as a survival advantage. The resulting CARs were named “second or third generation CARs”^38^. Many studies have verified that third generation CARs that combined the CD134 or CD137 intracellular co-stimulatory domain with CD28 and CD3ζ modules exhibited enhanced persistence and antitumour activity compared with the second generation CARs containing only CD28 and CD3ζ signal domains in tandem^39, 40^. However, a recent study showed that second generation CAR (anti-hCEA-28Z)-transduced CIK cells exhibited superior antitumour efficacy to those engineered with a third generation CAR (anti-CEA-28-OX40-Z), which was mainly due to accelerated maturation and the enhanced activation-induced cell death (AICD) effect of the latter^41^. This discrepancy indicated that the optimal combination of intracellular signal domains might vary and empirically rely on each individual study^42^. Because we used CIK cells for CAR gene modification, the second generation CAR seemed to be a better choice. Based on the confirmed information, we successfully generated 5T4-28Z CAR and C-28Z CAR-transduced CIK cells. The CAR gene transduction did not alter the typical phenotypes of CIK cells. This result was consistent with related findings in several previous studies^43, 44^.

Functional assays, including LDH release, IFN-γ secretion and CD107α expression, demonstrated that the 5T4-28Z-engineered CIK cells showed both CAR-redirected and CAR-independent cytolytic activity against NPC *in vitro*. Particularly, the 5T4-28Z-CIK cells were capable of efficiently attacking 5T4^high^ spheroid cells which likely represent putative CSCs in NPC. Notably, the CAR gene modification of CIK cells did not inhibit their original MHC-unrestricted antitumour activity which is principally mediated by the interaction between NKG2D and stress-inducible molecules such as MHC class I-related chain A and B (MIC A/B) and UL-16-binding proteins (ULBPs) expressed on the surface of tumour cells^10^. However, in contrast to the results of the cytotoxicity and cytokine release assays, we noticed that the cytotoxic granule release of 5T4-28Z-CIK cells was not inhibited by NKG2D blocking. This finding was inconsistent with the results of a previous study showing that stimulation of NKG2D did augment CD107α expression^9^. This discrepancy might be attributed to the fact that those researchers examined promotion of CD107α expression on non-transduced CIK cells after receiving an NKG2D activation signal, while this effect might be covered up by the CAR which endowed CIK cells with a more potent activation signal when encountering antigen even at a low level. In addition, over expression of 5T4 on 5T4^dim/-^ NPC cells dramatically promoted the activation of 5T4-28Z-CIK cells and in turn rendered them capable of killing 5T4-expressing cells (Supplementary Fig. S3). This finding was similar to those in related studies^34, 35^ and further demonstrated the antigen specificity of 5T4-28Z-CIK cells. However, the effectiveness of our CAR-CIK cells *in vivo* still needs to be tested in animal models.

In summary, our study revealed that the oncofetal antigen 5T4 was restrictively expressed on the surface of NPC cells and is associated with the stemness of NPCSCs, which makes it a promising target for CAR-redirected CIK-cell-based immunotherapy against NPC. We successfully generated a 5T4-28Z CAR-encoded lentiviral vector and produced 5T4-28Z CAR-engineered CIK cells. We also showed that these gene-modified cells possessed both CAR-redirected and CAR-independent anti-NPC activity and could efficiently eliminate NPC cells, particularly NPC stem cell-like cells *in vitro*.

## Methods

### Plasmids and antibodies

The lentiviral vector pHAGE-fullEF1α-MCS-IRES-ZsGreen and the lentiviral packaging plasmids psPAX2 and pMD2.G were all kindly provided by Prof. Didier Trono (University of Geneva, Geneva, Switzerland). The following antibodies were used: anti-hROR1 polyclonal antibody (R&D), anti-hROR1-APC antibody (R&D), anti-h5T4-APC antibody (R&D), anti-h5T4 mAb (clone EPR5529, Abcam), anti-E-cadherin mAb (clone EP700Y, Abcam), anti-human N-cadherin mAb (clone EPR1791-4, Abcam), anti-human Nanog mAb (clone EPR2027(2), Abcam), anti-human Oct4 mAb (clone EPR2054, Abcam), anti-human vimentin mAb (clone EPR3776, Abcam), anti-human sox2 mAb (clone EPR3131, Abcam), anti-human β-catenin mAb (clone E247, Abcam), anti-human Carbonic Anhydrase IX polyclonal antibody (GeneTex), anti-human GAPDH antibody (Proteintech), HRP-conjugated secondary antibodies (Proteintech), diverse fluorochrome-conjugated anti-human CD3, CD8, CD56, NKG2D, CD57, PD-1, CD107α and isotype control antibodies (Pharmingen, BD).

### Description of clinical tissue samples

All investigations were performed in accordance with the Declaration of Helsinki, ethical standards and national/international guidelines, and with approval from the Guangdong Medical College Institutional Research Ethics Committee. The 60 NPC tissues and 14 inflammatory nasopharyngeal tissues were collected from the Department of Pathology, People’s Hospital of Gaozhou City, Affiliated Hospital of Guangdong Medical College, China, during the period from 2003 to 2005. All patients in this study signed an informed consent and had received neither radiotherapy nor chemotherapy before tissue biopsies were obtained. According to the criteria of the WHO histological classification (2005), all NPC samples were diagnosed as non-keratinizing squamous cell carcinoma, including 9 cases of differentiated non-keratinizing carcinoma and 51 cases of undifferentiated non-keratinizing carcinoma.

### Immunohistochemistry

Formalin-fixed, paraffin-embedded tissue sections (3 μm) were heated at 60 °C for at least 2 h prior to being de-paraffinized in xylene and rehydrated in an ethanol to distilled water gradient. For antigen retrieval, slides were immersed into citrate buffer and maintained in heat for 3 min under high pressure. After the endogenous peroxidase activity and nonspecific binding was blocked with 3% H_2_O_2_ and 10% goat serum, respectively, the slides were incubated with appropriately diluted specific primary antibodies overnight at 4 °C. Subsequently, the slides were further incubated with biotinylated secondary antibodies and HRP-conjugated streptavidin, followed by signal detection using a DAB plus Kit (DAKO) and counterstaining with haematoxylin. Finally, the slides were dehydrated in graded ethanol, mounted with Neutral balsam and examined under a bright field microscope.

The IHC results were evaluated by two pathologists independently and blindly. In each case, four random areas were selected and assessed at 400× magnification. Both the intensity of immunostaining and the proportion of positively stained tumour cells were used to quantify the IHC result^45^. Staining intensity was defined as either 0, no staining; 1, light yellow staining; 2, yellow staining; or 3, brown staining. Likewise, the proportion of stained cells was quantified using four grades: 0, no positively stained tumour cells; 1, < 10%; 2, 10~50%; 3, > 50%. Membranous and cytoplasmic expression levels depended on the product of the two parts. Final scores of ≤4 and ≥6 were considered to be tumours with weak and strong expression, respectively.

### Tumour sphere formation assay

NPC cells in logarithmic growth phase were collected, counted and seeded into Ultra Low Attachment six-well plates (Corning) at a density of 1,000 cells per well in triplicate. Then, the cells were cultured in serum-free mammary epithelial cell basal medium (MEBM, Lonza) supplemented with 20 ng/ml EGF (Peprotech), 20 ng/ml bFGF (Peprotech) and 1 × B27 supplement (Gibco) at 37 °C in a humid atmosphere with 5% CO_2_. One week later, the diameters of tumour spheroids were measured and those that were ≥ 100 μm in diameter were counted under a bright field microscope.

### Generation of CAR constructs and preparation of CAR-encoded lentivirus

The VH and Vκ derived from a mouse mAb specific for human 5T4^31^ were fused with a (Gly_4_Ser) _3_ linker, and the resulting scFv was applied to design the anti-h5T4 CAR construct. A DNA sequence encoding the CAR was designed from the 5′ end to the 3′ end according to the pattern below: CD8α SP (AA, 1~21), scFv, myc-tag, CD8α hinge (AA, 136~182), the transmembrane and intracellular portion of human CD28 (AA, 153~220) and the cytoplasmic domain of human CD3ζ (AA, 52~163). The entire nucleotide sequence encoding the anti-h5T4 CAR (5T4-28Z) was codon-optimized, synthesized and ligated into the NheI and XbaI sites of a pHAGE-fullEF1α-MCS-IRES-ZsGreen lentiviral vector. Likewise, a similar CAR (C-28Z) structure that lacked the scFv region compared with 5T4-28Z was also constructed and cloned into the same vector as a negative control. Vectors encoding 5T4-28Z or C-28Z were verified by sequencing and, together with pMD2.G and psPAX2 plasmids, were co-transfected into HEK293T cells using Lipofectamine3000 reagent (Invitrogen) to generate lentivirus. The supernatants containing lentiviral particles were harvested at 48 h and 72 h after transfection, filtered through 0.45 μm cellulose acetate filters (Millipore) and then concentrated using ultra-centrifugation (Amicon^®^ Ultra-15 100 kDa, Millipore). Finally, the lentivirus titers were determined using a Lenti-X p24 Rapid Titer Kit (Clonetech) according to manufacturer’s protocol.

### Generation of CIK cells, transduction of CAR-encoded lentivirus and cell sorting

Human PBMCs were isolated from blood samples of healthy donors using density gradient centrifugation with Ficoll-Hypaque (TAKARA). To generate CIK cells, PBMCs were initially cultured at a density of 2 × 10^6^ cells/ml in serum-free X-Vivo15 medium (Lonza) supplemented with IFN-γ (1,000 U/ml, Peprotech). After 24 h, anti-CD3 mAb (OKT3, 100 ng/mL, eBioscience), anti-CD28 mAb (100 ng/mL, eBioscience) and IL-2 (500 U/ml, Peprotech) were added. After an additional 48 h of cultivation, 5 × 10^5^ CIK cells were mixed with 400 ng of concentrated lentiviral particles, seeded into a 24-well cell culture plate and centrifuged at 1,000 g for 1 h in the presence of protamine sulfate (8 μg/ml, Sigma). Then, the plate was incubated for 18 h at 37 °C under 5% CO_2_ before the supernatant was replaced with fresh complete medium containing 500 U/ml of IL-2. After 72 h, the GFP-positive cells were sorted using a BD FACSAria^TM^ IIu flow cytometer. An anti-myc-tag-Alexa Fluor^®^ 647 antibody (clone#9B11, CST) was used to identify CAR expression on the surface of CIK cells. CAR-expressing CIK cells and NT-CIK cells were propagated at a density of 2 × 10^6^ cells/ml for 2~3 weeks, and cultures were supplemented with fresh medium every 2~3 days. Phenotypic analysis was performed weekly using FACS.

### Functional assays (LDH release, IFN-γ secretion, degranulation)

The target cells used in these assays included S18 cells, S26 cells and their spheroid cells. Before experiments, tumour spheroids were collected using a 100 μm cell strainer (BD) and dissociated to single cells with treatment of Tryple Express (Gibco). Effector cells included 5T4-28Z-CIK, 5T4-28Z-CIK (NKG2D-), C-28Z-CIK and NT-CIK cells, which were all harvested during 2~3 weeks of culture. For the NKG2D blocking group, 5T4-28Z-CIK cells were pre-incubated with 20 μg/ml of anti-NKG2D neutralizing antibody (clone #149810, R&D) for 30 min at 37 °C before co-cultivation with target cells.

Cytotoxicity assays were performed in U-bottom 96-well plates in a final sample volume of 100 μl per well. Briefly, 1 × 10^4^ target cells per well were co-cultured with different effector cells at E/T ratios of 10/1, 5/1 and 2.5/1 for 4 h. All samples were set in triplicate. Next, 50 μl of supernatant per well was collected to measure LDH release using a cytotoxicity LDH Assay Kit-WST^®^ (Dojindo) according to the manufacturer’s specifications. The cell lysis percentage was calculated as follows: Cytotoxicity (%) = (Experimental – Effector Spontaneous – Target Spontaneous) / (Target Maximum – Target Spontaneous) × 100.

In IFN-γ secretion assays, 2 × 10^4^ target cells per well were co-incubated with 1 × 10^5^ effector cells per well in 96-well U-bottom plates in triplicate in a total volume of 200 μl/well. After 24 h, cell-free supernatants were harvested for analysis of IFN-γ production using a Human IFN-γ ELISA Ready-SET-Go Kit (eBioscience) in accordance with the manufacturer’s protocol.

In degranulation assays, 2 × 10^5^ target cells per well were co-cultured with 1 × 10^6^ effector cells per well at 37 °C for 5 h in the presence of Protein Transport Inhibitor Cocktail (1×, eBioscience) and anti-human CD107α-APC antibody or isotype control antibody (Pharmingen, BD). All samples were set in triplicate. Cells were then harvested, washed and re-suspended in PBS. Staining results were analysed using FACS.

### Time-lapse imaging

NPC cells in logarithmic growth phase were harvested, stained with the red fluorescent dye CM-Dil (Sigma) and induced to spheroids in tumour sphere medium, as mentioned above. After five days of culture, tumour spheroids were sorted and co-cultured with 5T4-28Z-CIK cells in a tissue culture plate and monitored using an inverted fluorescence microscope with climate control (Zeiss Observer Z1). In another group, the interaction between CM-Dil-labelled NPC cells and effector cells was examined in the same way. Image capture started from the beginning of the co-incubation with an interval time of 3 min and did not stop until the tumour spheroids dissociated and disappeared. Data were processed using ZEN2011 software (Zeiss).

### Statistical analysis

Statistical analysis was performed using GraphPad Prism^®^ software v5.0 (GraphPad) and Statistical Package for Social Sciences (SPSS) software v20.0. All experiments were repeated at least three times, and all experimental data in figures and text are shown as the mean ± standard deviation. Two-tailed Student’s t-test was used to compare two normally distributed independent groups with continuous endpoints. One-way ANOVA followed by Dunnett’s post hoc test was used when more than two independent groups were compared. A standard χ^2^ test was applied to compare the target gene expression between NPC and non-cancerous nasopharyngeal tissue specimens. *p* values less than 0.05 were considered statistically significant.

## Electronic supplementary material

Supplementary Video S1

Supplementary Video S2

Supplementary Information
