# Supplementary material for: 5T4-specific chimeric antigen receptor modification promotes the immune efficacy of cytokine-induced killer cells against nasopharyngeal carcinoma stem cell-like cells
Source: Sci Rep. 2017 Jul 7;7:4859. doi: 10.1038/s41598-017-04756-9 (PMC5501797; doi:10.1038/s41598-017-04756-9)
Supplement: Supplementary file 3 — Supplementary Information [file 41598_2017_4756_MOESM3_ESM.pdf]

Supplementary Information for

"5T4-specific chimeric antigen receptor modification promotes the immune efficacy of cytokine-induced killer cells against nasopharyngeal carcinoma stem cell-like cells"

Xueyang Guo[1], Hang Zheng[2], Weiren Luo[1, 3], Qianbing Zhang[1], Jingxian Liu[1] and Kaitai Yao[1,*]

[1]Guangdong Provincial Key Laboratory of Cancer Immunotherapy and Guangzhou Key Laboratory of Tumour Immunology Research, Cancer Research Institute, Southern Medical University, Guangzhou, China; [2]Department of Oncology, Nanfang Hospital, Southern Medical University, Guangzhou, China; [3]Department of Pathology, Shenzhen Third People's Hospital, Shenzhen University, Shenzhen, China

* Corresponding author: Kaitai Yao

E-mail: yao.kaitai@hotmail.com

Work telephone Numbers: +86 20 6164 8225

Postal address: Room 308, Life-Science Building, Southern Medical University, No.1023, Shatai South Road, Baiyun District, Guangzhou City, Guangdong Province, China

Postal code: 510515

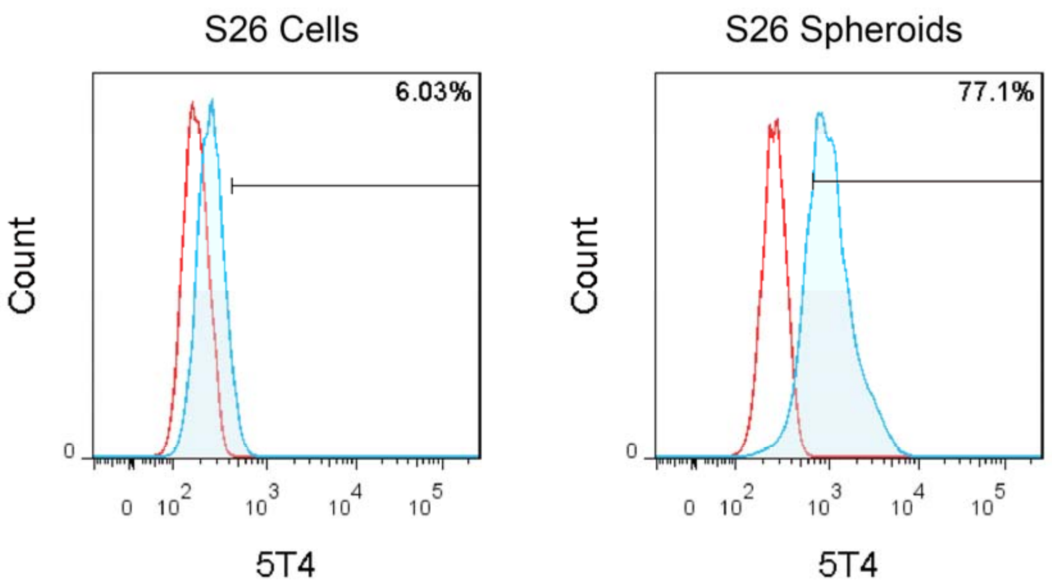

Supplementary Figure S1. Flow cytometry analysis of membranous 5T4 expression on S26 spheroid cells and the parental cells. Cells were stained with APC-conjugated anti-h5T4 antibody or matched isotype control antibody. Typical flow plots are shown.

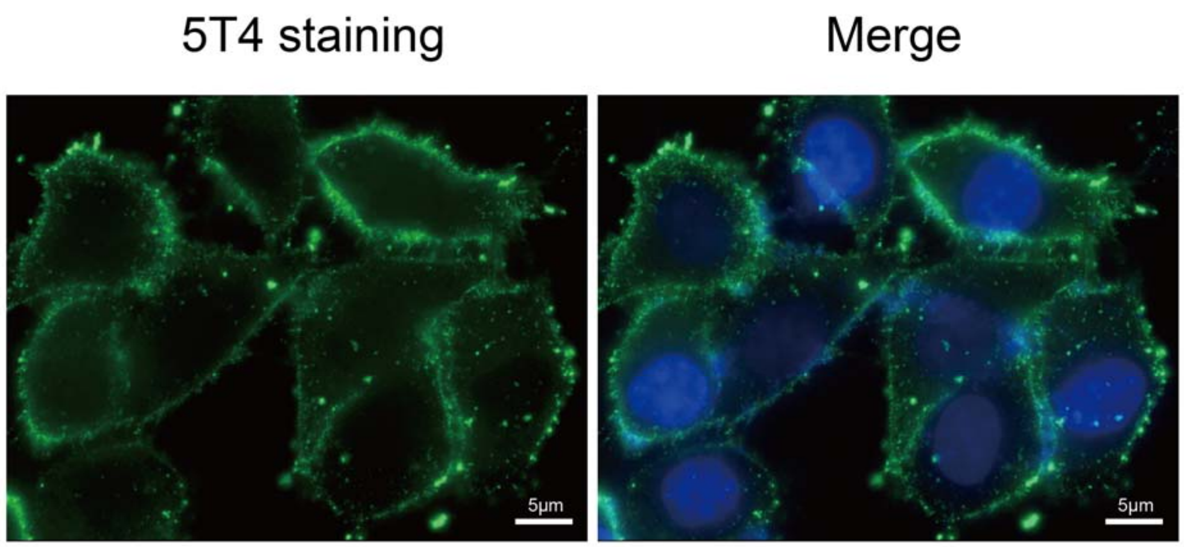

Supplementary Figure S2. Immunofluorescence analysis of the cellular distribution of 5T4 in NPC. SUNE1 cells were stained with 5T4 primary antibody followed by FITC-conjugated anti-rabbit secondary antibody and counterstained with DAPI. A typical image of 5T4 staining (a) and a merged picture (b) are shown.

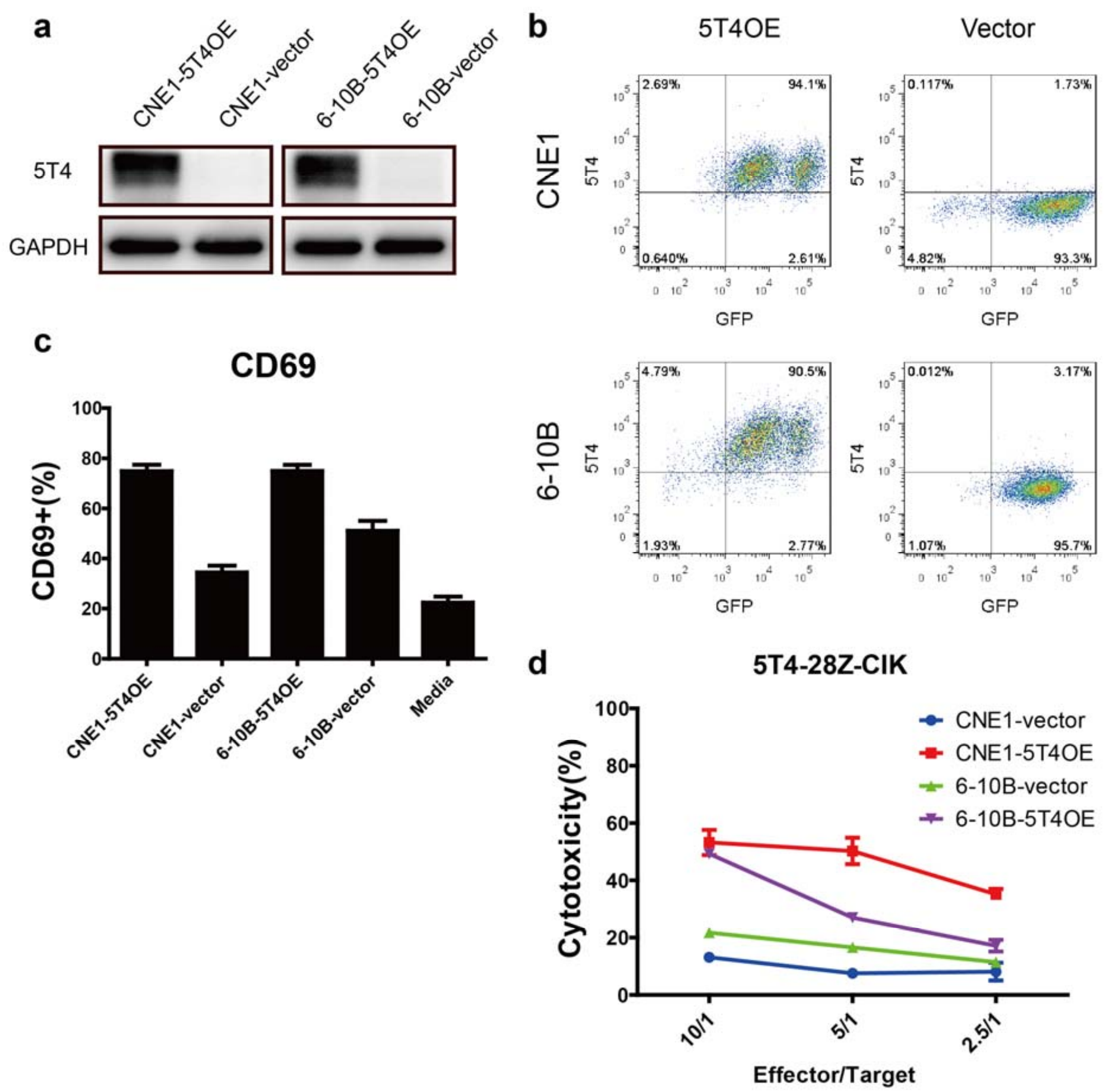

Supplementary Figure S3. Over-expression of 5T4 on NPC cells promotes the activation and cytotoxicity of 5T4-28Z-CIK cells. Stable transduction of 5T4 in 6-10B and CNE1 cell lines was confirmed using WB (a) and flow cytometry (b) with anti-h5T4 antibody. (c) Then, 2×10^5 target cells per well were co-cultured with 1×10^6 5T4-28Z-CIK cells per well for 24 h. Effector cells cultured alone served as the negative control. The expression of CD69 on effector cells was detected with flow cytometry using anti-hCD69-APC antibody. Values in the histogram represent the mean ± SD of three parallel samples. (d) Next, 1×10^4 target cells per well were co-cultured with 5T4-28Z-CIK cells at E/T ratios of 10/1, 5/1 and 2.5/1 for 4 h. The cytotoxicity of effector cells was examined with LDH release assays. Values in the line graphs represent the mean ± SD of three parallel wells.

Supplementary Video S1. 5T4-28Z-CIK cells efficiently eliminate S26 NPC cells. The video corresponds to the images displayed in Fig. 6g.

Supplementary Video S2. 5T4-28Z-CIK cells discern and attack S26 Spheroid. The video corresponds to the images displayed in Fig. 6g.

Supplementary methods

Cell lines. Seven human NPC cell lines without Epstein-Barr virus (EBV) infection, including CNE1; SUNE1 and its sub-clones 5-8F, 6-10B; CNE2 and its sub-clones S18, S26; and the normal nasopharyngeal epithelial cell line NP69, were kindly provided by Prof. M.-S. Zeng (Sun Yat-sen University, Guangzhou, China). The EBV-positive NPC cell line HONE-1 was obtained from Prof. S.-W. Tsao (University of Hong Kong, Hong Kong, China). The NP69 cell line was maintained in keratinocyte serum-free medium (Gibco). All NPC cell lines and HEK293T cells were cultured in RPMI-1640 medium (Gibco) or DMEM (Gibco) supplemented with 10% FBS (HyClone) at 37℃ under 5% CO_2.

RNA extraction, cDNA synthesis and real-time quantitative PCR. The total RNA was extracted from cells using an RNAiso Plus kit (TAKARA) according to the manufacturer’s protocol. Each cDNA was synthesized from 500 ng of total RNA using a PrimeScript™ RT reagent Kit (TAKARA) in accordance with the manufacturer’s instructions. Then, each 10 ng of cDNA was used as a template for PCR amplification with an SYBR® Premix Ex Taq™ II kit (TAKARA). The PCR was performed for 40 cycles of 5 s at 95℃ and 35 s at 60℃, using an Mx3000PTM real-time PCR system (Stratagene). The level of gene expression was determined by the comparative cycle threshold method ($2^{-\Delta Ct}$), in which *GAPDH* was used as the internal reference. Special primer pairs designed by Primer

Premier 6 (Premier Biosoft) were used and are shown as follows: *ROR1*-F, 5’-CAGTCAGTGCTGAATTAGTGCC-3’; *ROR1*-R, 5’-TCATCGAGGGTCAGGTAAGAAT-3’; *5T4*-F, 5’-CGCTACTCTGGTGGAACTCA-3’; *5T4*-R, 5’-ACCTCTTCGCCTCTTGTTGG-3’; *CAIX*-F, 5’-CTGCTGGTGACATCCTAGCC-3’; *CAIX*-R, 5’-CTCCAGTCTCGGCTACCTCT-3’; *GAPDH*-F, 5’-ACAACTTTGGTATCGTGGAAGG-3’; and *GAPDH*-R, 5’-GCCATCACGCCACAGTTTC-3’.

Western blotting. NPC cells and their spheroids were lysed in RIPA lysis buffer (Beyotime) supplemented with 1% protease inhibitor cocktail (Pierce) and incubated for 30 min on ice. Then, the suspensions were collected and centrifuged at 12,000 g and 4℃ for 5 min, and the protein concentration of the supernatants was determined using a BCA protein assay kit (Beyotime) according to the operating manual. The cell lysates (30 μg per lane) were separated on 10% polyacrylamide gels by SDS-PAGE under reducing conditions and then transferred onto a 0.45 μm PVDF membrane (Millipore). Then, the membrane was blocked with 5% BSA-TBS-Tween 20 at room temperature for 1 h and incubated with the appropriately diluted specific primary antibodies overnight at 4℃. After five washes with 0.1% TBS-Tween 20, the membranes were incubated with HRP-conjugated secondary antibodies at room temperature for 2 h, followed by five additional washes with 0.1% TBS-Tween 20. Finally, the bands were detected using a ChemiDoc XRS+ imaging system (Bio-Rad) with a BeyoECL Star Kit (Beyotime).

Flow cytometric analysis. For cell surface staining, 10^6 cells were washed with PBS and resuspended in 100 μl of 2% FBS-PBS. Then, fluorochrome-conjugated antibodies or matched isotype control antibodies were added into the suspension and incubated at 4℃ for 30 min. Subsequently, the cells

were washed twice with 2% FBS-PBS prior to analysis on BD LSRFortessa^{TM} system. Flowjo software v7.6.1 (Tree star) was used for data analysis.

Immunofluorescence. SUNE1 cells seeded on coverslips were washed with PBS and fixed with 4% paraformaldehyde for 20 min. Then, the coverslips were blocked with 5% BSA-PBS for 30 min at room temperature and incubated with rabbit anti-h5T4 antibody (1/100, clone EPR5529, Abcam) overnight at 4℃. The cells were then rinsed in PBS containing 0.05% Tween-20 three times and stained with FITC-conjugated goat anti-rabbit secondary antibody (1/100, BD) for 1 h in the dark at room temperature. After three washes in 0.05% PBS-T, the cells were counterstained with 4',6-diamidino-2-phenylindole (DAPI, Beyotime) and preserved in the mounting medium. Images were captured under an inverted fluorescence microscope (Zeiss Observer Z1) with a 63×/1.4 numerical aperture oil immersion objective, and representative pictures from Z-axis sections were overlapped using ZEN2011 software (Zeiss). For the negative control, coverslips were incubated in 1% BSA-PBS instead of primary antibody.

Generation of 5T4 stably expressing NPC cells. Human 5T4 cDNA was cloned into a pHBLV-CMVIE-ZsGreen-T2A-Luciferase vector and transduced into 5T4$^{dim/-}$ 6-10B cells and 5T4$^{dim/-}$ CNE1 cells using lentivirus. The stably transduced cells were sorted using FACS according to the GFP expression, and expression of 5T4 was verified using WB and flow cytometry with anti-h5T4 antibody. As the negative controls, 6-10B and CNE1 cells were transduced with vacant vectors.

Functional assays. In the CD69 assay, 2×10^5 target cells (6-10B-5T4OE, 6-10B-vector, CNE1-5T4OE, CNE1-vector) per well were co-cultured with 1×10^6 5T4-28Z-CIK cells per well at 37℃ under 5% CO_2. All samples were set in triplicate. After incubation for 24 h, cells were collected,

stained with anti-CD69-APC antibody or matched isotype control antibody (Pharmingen, BD) and monitored using FACS. A cytotoxicity assay was performed in the same manner as mentioned in the Methods section.
